# MicroRNA-17-5p aggravates lipopolysaccharide-induced injury in nasal epithelial cells by targeting Smad7

**DOI:** 10.1186/s12860-018-0152-5

**Published:** 2018-02-13

**Authors:** Nan Huang, Wenjing Li, Xiaolong Wang, Shanshan Qi

**Affiliations:** 1 Department of Allergy, Tongji Hospital, Tongji Medical College, Huazhong University of Science and Technology, Wuhan, 430030 China; 2 Department of Allergy, Wuhan No.1 Hospital, No. 215, Zhongshan Avenue, Wuhan, 430022 China

**Keywords:** LPS, miR-17-5p, RPMI2650, Smad7, CCK-8 assay, Rhinitis

## Abstract

**Background:**

Globally, rhinitis is one of the most common chronic disorders. Despite availability of drugs to manage the symptomatology of rhinitis, researchers still focus on identification of novel molecular targets for better management. MicroRNAs are implicated in many biological and pathological processes. However, the role of miR-17-5p in rhinitis remains unexplored. This study aimed to explore the role of miR-17-5p in lipopolysaccharide (LPS)-induced injury of nasal epithelial RPMI2650 cells and to elucidate the possible underlying molecular mechanism.

**Results:**

LPS damaged RPMI2650 cells by inhibiting cell proliferation, promoting apoptosis, and stimulating the release of inflammatory cytokines. miR-17-5p expression was significantly increased in RPMI2650 cells following treatment with LPS. Furthermore, it was found that overexpression of miR-17-5p led to aggravation of LPS-induced injury. miR-17-5p negatively regulated expression of Smad7; overexpression of Smad7 protected the RPMI2650 cells by inactivating NF-κB and Wnt/β catenin pathways and vice versa.

**Conclusions:**

Overexpression of miR-17-5p aggravated LPS-induced damage of RPMI2650 cells. Expression of Smad7 was negatively regulated by miR-17-5p; Smad7 expression inactivated NF-κB and Wnt/β catenin pathways.

## Background

Rhinitis is one of the most common inflammatory disorders of the upper airways [1]. This condition is triggered by exposure of the nasal mucosal cells to allergens. Current statistics suggest that approximately 15% of the adolescents are suffering from allergic rhinitis worldwide [1, 2]. In addition to nasal obstruction, sense of itching and frequent sneezing, rhinitis is also one of the important causes of disturbed sleep [1]. This condition is difficult to diagnose in young children [3]. Identification of the possible genetic and environmental mutagenicity factors, elucidation of the molecular pathways implicated in the pathogenesis of rhinitis, identification of novel drug targets, and improvement of current treatment strategies, remain the principal goal in rhinitis research [1–3].

MicroRNAs (miRNAs or miRs) belong to the family of non-coding RNAs, as their name suggest, they are smaller in size, consisting of 22–25 nucleotides. miRNAs bind to the 3’-UTR (untranslated region) of their corresponding mRNA and cause post-translational inhibition of these mRNAs [4]. miRNAs are known to be expressed widely in human body and they modulate diverse physiological and pathological processes like organ development, cell proliferation, cell differentiation, tumorigenesis, and apoptosis [5]. Studies have already established the role of several miRNAs in rhinitis, including miR-21, miR-30-5p, miR-199b-3p, miR-874, miR-28-3p, miR-203, miR-875-5p, etc. [6–8]. Some of the above mentioned miRNAs are high expressed while some are low expressed [6–8].

Several studies have explored the role of miR-17-5p in different cancers [9–12]. For instance, miR-17-5p mediated hypoxia-induced autophagy and inhibited apoptosis in vascular smooth muscle cells [13]. Increased miR-17-5p expression induced proliferation and inhibited apoptosis of lung cancer cells, while reduced lung cancer cell sensitivity to Gefitinib [14]. Besides, miR-17-5p has been considered as a potential therapeutic target for atherosclerotic lesions [15], retinal inflammation [16], non-traumatic osteonecrosis of femoral head [17], and fatty liver [18]. However, no study has been carried out to explore the role of miR-17-5p in rhinitis.

Lipopolysaccharide (LPS), a toll-like receptor 4 agonist, is the major cell wall component of Gram-negative bacteria. Its principal function is to maintain structural integrity of the bacterial cell [19]. LPS also acts as an endotoxin that produces strong immune response and inflammation [20]. Studies have already used LPS-induced nasal epithelial cell damage as rhinitis model [19]. In this study we have explored the role of miR-17-5p in LPS-induced nasal epithelial cell damage and also tried to explore the underlying molecular pathways and targets.

## Methods

### Cell culture and treatment

Human nasal epithelial cell line (RPMI2650) was purchased from American Type Culture Collection (ATCC, Rockville, MD, USA). RPMI2650 cells were routinely cultured in RPMI 1640 (Invitrogen, Carlsbad, CA, US) supplemented with 10% fetal bovine serum (FBS; Sigma, St. Louis, MO, USA) in presence of penicillin/streptomycin (Sigma, St. Louis, MO, USA) at 37 °C in a humidified chamber with 5% CO_2_. The cells were treated by LPS (5 μg/mL) for 12 h.

### miRNAs transfection

Scramble, siNC, si-miR-17-5p, and miR-17-5p mimic were synthesized by GenePharma Co (Shanghai, China). Cell transfections were conducted using Lipofectamine 3000 reagent (Invitrogen) as per the manufacturer’s protocol.

### Quantitative real-time PCR (RT-PCR)

RNAs from the cultured cells were extracted using RNA pure Rapid Extraction Kit (Bioteke Corporation, Beijing, China) according to the manufacturer’s instructions. For reverse transcription of miRNA, single-step cDNA synthesis was done by adding poly (A) tail to the 3′ end of miRNAs with oligo (dT) adaptor primer and Super M-MLV reverse transcriptase (Bioteke Corporation, Beijing, China). For mRNA, total RNAs were reversely transcribed in a reaction system containing random primers and M-MLV reverse transcriptase. Subsequently, the reverse transcription products (cDNA) were amplified by using real-time polymerase chain reaction (RT-PCR) with SYBR green Master Mix; RT-PCR was performed in Exicycler 96 Real-Time Quantitative Thermal Block (BIONEER, Daejeon, South Korea). U6 was used as the internal control for miRNA expression analysis, while GAPDH was used as the internal control for determination of mRNA expression levels. The RT-PCR conditions were as follows: initial 10 min incubation at 95 °C, then 40 cycles at 95 °C for 10 s, at 60 °C for 20 s, and at 72 °C for 30 s, followed by 5 min incubation at 4 °C. Relative quantification analysis was conducted using the 2^−△△CT^ method. Each sample was analyzed in triplicate, and all experiments were carried out three times independently.

### Transfection and generation of stably transfected cell lines

Full-length Smad7 sequences and short-hairpin RNA directed against Smad7 were constructed in pEX-2 and U6/GFP/Neo plasmids (GenePharma), respectively. They were referred to as pEX- Smad7 and sh- Smad7, respectively. The lipofectamine 3000 reagent (Life Technologies Corporation, Carlsbad, CA, USA) was used for the cells transfection according to the manufacturer’s instructions. The plasmid carrying a non-targeting sequence was used as a negative control (NC) of sh-Smad7 referred to as sh-NC. The stably transfected cells were selected by using culture medium containing 0.5 mg/mL G418 (Sigma-Aldrich, St Louis, MO, USA). After approximately 4 weeks, G418-resistant cell clones were established.

### CCK-8 assay

Cells were seeded in 96-well plate with 5000 cells/well. Cell viability was assessed by a Cell Counting Kit-8 (CCK-8, Dojindo Molecular Technologies, Gaithersburg, MD). Briefly, after stimulation, the CCK-8 solution was added to the culture medium, and the cultures were incubated for 1 h at 37 °C in humidified 95% air and 5% CO_2_. The absorbance was measured at 450 nm using a Microplate Reader (Bio-Rad, Hercules, CA).

### Apoptosis assay

Apoptosis analysis was performed to identify and quantify the apoptotic cells by using Annexin V-FITC/PI apoptosis detection kit (Beijing Biosea Biotechnology, Beijing, China). The cells (100,000 cells/well) were seeded in 6 well-plate. Treated cells were washed twice with cold PBS and resuspended in buffer. The adherent and floating cells were combined and treated according to the manufacturer’s instruction and measured with flow cytometer (Beckman Coulter, USA) to differentiate apoptotic cells (Annexin-V positive and PI-negative) from necrotic cells (Annexin-V and PI-positive).

### Elisa

Culture supernatant was collected from 24-well plates and concentrations of inflammatory cytokines measured by enzyme-linked immunosorbent assay (Elisa) using protocols supplied by the manufacturer (R&D Systems, Abingdon, UK).

### Cytotoxicity assay

The cytotoxicity was tested by using the LDH Cytotoxicity Assay Kit (Beyotime, Shanghai, China). In brief, cells were seeded in 96-well plate with 5000 cells/well, and growth to 80~ 90% confluence. Supernatant of each well (50 μl) was transferred to a clear 96-well plate and 100 μl Reaction Mixture was added into each well. After 30 min of incubation at room temperature, the absorbance at a wavelength of 450 nm was determined using an Elisa instrument.

### Dual luciferase activity assay

The 3’UTR target site was generated by PCR and the luciferase reporter constructs with the Smad7 3’UTR carrying a putative miR-17-5p-binding site into pMiR-report vector were amplified by PCR. Cells were co-transfected with the reporter construct, control vector, and miR-17-5p or scramble using Lipofectamine 3000 (Life Technologies, USA). Reporter assays were done using the dual-luciferase assay system (Promega) following the manufacturer’s information.

### Western blot

Proteins used for western blotting were extracted using RIPA lysis buffer (Beyotime Biotechnology, Shanghai, China) supplemented with protease inhibitors (Roche, Guangzhou, China). The proteins were quantified using the BCA™ Protein Assay Kit (Pierce, Appleton, WI, USA). The western blot system was established using a Bio-Rad Bis-Tris Gel system according to the manufacturer’s instructions. GAPDH antibody was purchased from Sigma. Primary antibodies were prepared in 5% blocking buffer at a dilution of 1:1000 for detection of Bcl-2 (ab196495), Bax (ab32503), caspase-3 (ab13586), caspase-9 (ab25758), Smad7 (ab90086), p-p65 (ab76302), p65 (ab16502), p-IkBα (ab133462), IkBα (ab7217), Wnt3a (ab169175), Wnt5a (ab72583), β-catenin (ab6302), and GAPDH (ab9485, Abcam, Cambridge, MA, USA). Primary antibodies were incubated overnight with the membrane at 4 °C, followed by washing and incubation with secondary antibodies (ab6721, and ab6789, Abcam) marked by horseradish peroxidase for 1 h at room temperature. After rinsing, the Polyvinylidene Difluoride (PVDF) membrane carrying the blots and the antibodies were transferred into the Bio-Rad ChemiDoc™ XRS system, and then 200 μl Immobilon Western Chemiluminescent HRP Substrate (Millipore, MA, USA) was added to cover the membrane surface. The signals were captured and the intensity of the bands was quantified using Image Lab™ Software (Bio-Rad, Shanghai, China).

### Statistical analysis

All experiments were repeated three times. The results of multiple experiments are presented as the mean ± standard deviation (SD). Statistical analyses were performed using Graphpad statistical software (GraphPad Software, San Diego, CA). *P*-values were calculated using a one-way analysis of variance (ANOVA). A P-value of < 0.05 was considered to indicate a statistically significant result.

## Results

### LPS induced cell injury and increased the expression of inflammatory cytokines in RPMI2650 cells

**Fig. 1:**
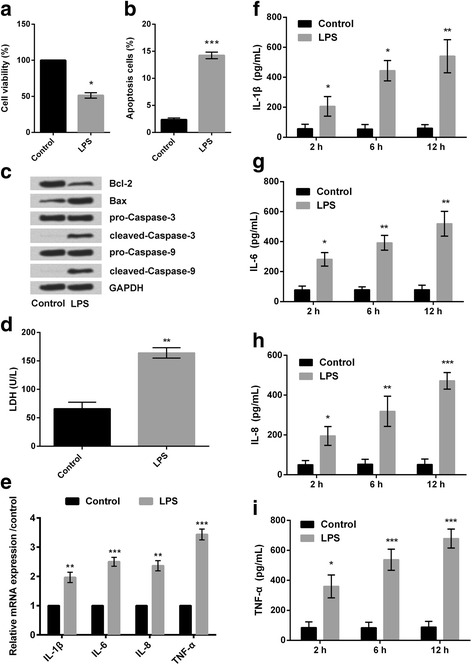
RPMI2650 cells were treated with LPS (5 μg/mL). **a** CCK-8 assay was done to estimate the percentage of viable cells in LPS-treated and control group of cells; **b** Flow cytometry was done to assess the percentage of apoptotic cells in LPS-treated and control groups of cells; **c** Western blot was done to estimate different apoptosis related proteins; **d** LDH cytotoxicity assay was done to detect the release of LDH from LPS-treated and control group of cells; **e** RT-PCR was to measure relative mRNA expression of different inflammatory cytokines; **f-i** Elisa was done to measure exact amount of different inflammatory cytokines, namely IL-1β, IL-6, IL-8, and TNF-α. LPS: lipopolysaccharide; CCK-8 assay: cell counting kit-8; RT-PCR: quantitative real time polymerase chain reaction; Elisa: enzyme linked immunosorbent assay; IL: interleukin; TNF-α: tumor necrosis factor α.*P < 0.05; **P < 0.01;***P < 0.001

CCK-8 assay revealed that following treatment of RPMI2650 cells with LPS (5 μg/mL); the percentage of viable cells was significantly decreased (*P* < 0.05; Fig. 1a) compared to the control group (not treated with LPS). Flow cytometry revealed that the percentage of apoptotic cells was significantly increased (*P* < 0.001; Fig. 1b) following treatment of RPMI2650 cells with LPS (5 μg/mL) compared to the control group of cells. Western blot analysis of the apoptosis-related proteins revealed: there was decreased expression of anti-apoptotic factor Bcl-2 and increased expression of pro-apoptotic factor like Bax, and other factors like cleaved-caspase-3, and cleaved-caspase-9 (Fig. 1c). LDH cytotoxicity assay results showed that the release of LDH was significantly increased in response to LPS when compared to the control group (*P* < 0.01, Fig. 1d).

Next, RT-PCR, revealed that the relative mRNA expression of the different inflammatory cytokines, including IL-1β, IL-6, IL-8, and TNF-α, were increased in the LPS treated cells (Fig. 1e) compared to the control group of cells. Similarly, actual estimation of the above mentioned inflammatory cytokines (done by Elisa) also revealed same results (Fig. 1f-i). Besides, it seems that LPS improved the release of inflammatory cytokines in a time-dependent manner. Considering that 12 h of LPS induced the most notably increases in inflammatory cytokine release, 12 h was selected as a LPS-stimulating condition for use in the following investigations.

### LPS induced expression of miR-17-5p

**Fig. 2:**
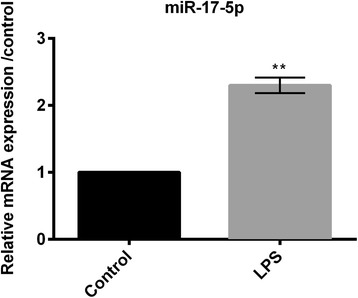
Expression of miR-17-5p in LPS-treated RPMI2650 cells was measured by RT-PCR. miR-17-5p: microRNA-17-5p; RT-PCR: quantitative real time polymerase chain reaction. **P < 0.01

Relative RNA expression of miR-17-5p (done by RT-PCR) revealed that the expression of miR-17-5p was significantly increased (*P* < 0.01; Fig. 2) in the LPS treated RPMI2650 cells compared to the control group of RPMI2650 cells.

### Overexpression and suppression of miR-17-5p in RPMI2650 cells

**Fig. 3:**
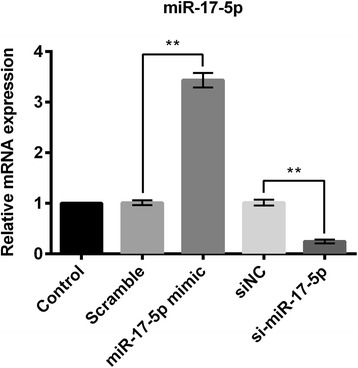
Following transfection of RPMI2650 cells with miR-17-5p mimic, scramble or si-miR-17-5p, expression of miR-17-5p was estimated in different groups of cells by RT-PCR. RT-PCR: quantitative real time polymerase chain reaction. **P < 0.01

Following transfection of RPMI2650 cells with scramble, siNC, si-miR-17-5p, and miR-17-5p mimic, RT-PCR was done to estimate the relative RNA expression of miR-17-5p. It was found that miR-17-5p expression was significantly increased in miR-17-5p mimic group of cells compared to the scramble group of cells. Similarly, miR-17-5p expression was significantly decreased in si-miR-17-5p group of cells compared to the siNC group of cells (*P* < 0.01; Fig. 3).

### Overexpression of miR-17-5p aggravated LPS-induced cell injury and the release of inflammatory cytokines

**Fig. 4:**
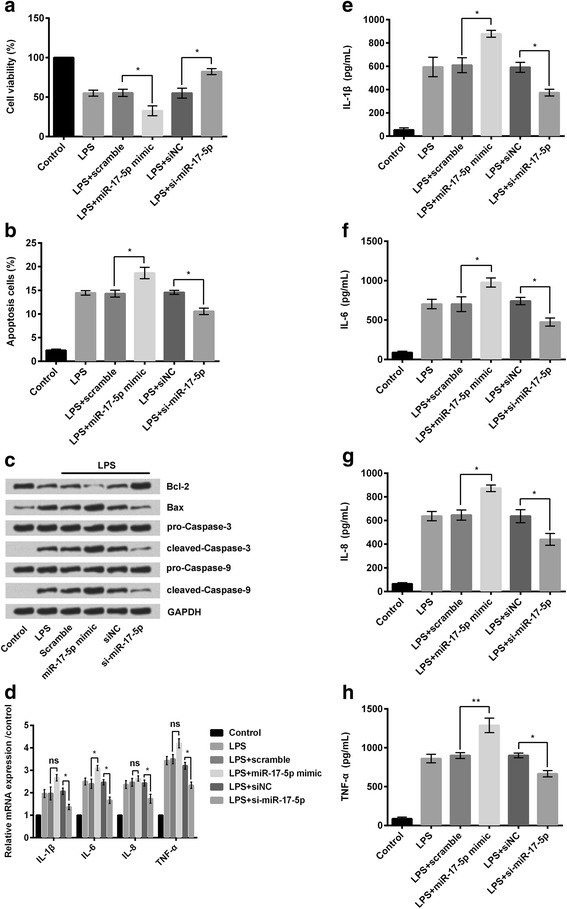
Following transfection of RPMI2650 cells with miR-17-5p mimic or si-miR-17-5p. **a** cell viability was assessed in different groups of cells; **b** Percentage of apoptotic cells in different group of cells were measured by flow cytometry; **c** Western blot analysis was done to assess different apoptosis related factors in different groups of cells; **d** Relative mRNA expression of different inflammatory cytokines (IL-1β, IL-6, IL-8, and TNF-α) were assessed by RT-PCR; **e-h** Elisa was done to measure the exact amounts of the said inflammatory cytokines in different groups of cells. Elisa: enzyme linked immunosorbent assay; IL: interleukin; TNF-α: tumor necrosis factor α.*P < 0.05; **P < 0.01

CCK-8 analysis expressed that the percentage of viable cells was significantly decreased (*P* < 0.05; Fig. 4a) following treatment with LPS (5 μg/mL) in RPMI2650 cells transfected with miR-17-5p mimic compared to the LPS-treated scramble group of cells. Similarly, in LPS-treated si-miR-17-5p group of cells, viability was significantly increased (*P* < 0.05; Fig. 4a) compared to the LPS-treated siNC group of cells. Flow cytometry revealed that the percentage of apoptotic cells was significantly increased (*P* < 0.05; Fig. 4b) in LPS-treated miR-17-5p mimic group of cells compared to the LPS-treated scramble group of cells. Similarly, apoptosis was significantly suppressed (*P* < 0.05; Fig. 4b) in LPS-treated si-miR-17-5p group of cells compared to the LPS-treated siNC group of cells. Western blot analysis revealed similar results as the amounts of pro-apoptotic factor Bax, cleaved-caspase-3, and cleaved-caspase-9, were increased in LPS-treated miR-17-5p mimic group of cells compared to other groups of cells, whereas treatment of si-miR-17-5p with LPS revealed opposite results (Fig. 4c). Next, RT-PCR was done to estimate the relative expression of different inflammatory cytokines, including IL-1β, IL-6, IL-8, and TNF-α (Fig. 4d) in different groups of cells. It was found that expressions of the inflammatory cytokines were increased (although not significantly) in the LPS-treated miR-17-5p mimic group of cells compared to the other group of cells, whereas expressions of the said inflammatory cytokines were minimum (not significant) in LPS-treated si-miR-17-5p group of cells (Fig. 4d). Elisa was done to estimate the amounts of the said inflammatory cytokines (Fig. 4h) released by the different groups of cells. Similar to Fig. 4c, it was found that the amounts of IL-1β (*P* < 0.05; Fig. 4e), IL-6 (P < 0.05; Fig. 4f), IL-8 (P < 0.05; Fig. 4g), and TNF-α (*P* < 0.01; Fig. 4h) released from the LPS-treated miR-17-5p mimic group of cells were significantly higher compared to the LPS-treated scramble group of cells. Similarly, knockdown of miR-17-5p as in si-miR-17-5p group of cells led to significant decrease (*P* < 0.05; Fig. 4e-h) in the amounts of the said inflammatory cytokines despite treatment with LPS.

Thus it was found that overexpression of miR-17-5p aggravated LPS-induced injury of RPMI2650 cells by suppressing cellular proliferation, promoting apoptosis, and facilitating release of inflammatory mediators.

### miR-17-5p negatively regulated expression of Smad7

**Fig. 5:**
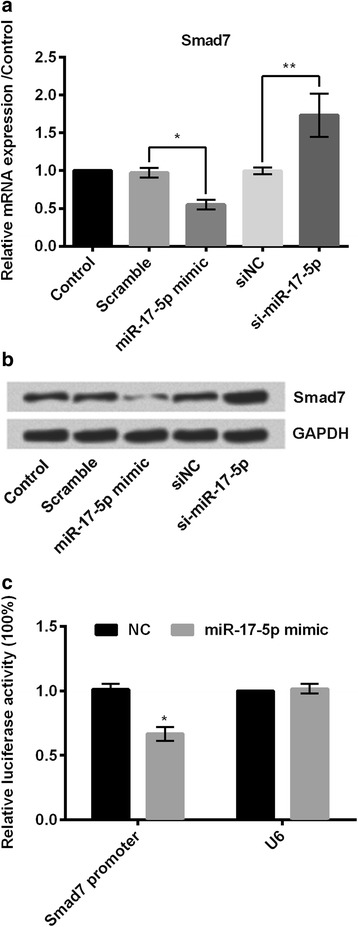
miR-17-5p negatively regulated expression of Smad7. miR-17-5p negatively regulated expression of Smad7 as in by **a** RT-PCR; **b** western blot analysis; and **c** relative luciferase activity. *P < 0.05; **P < 0.01

Relative mRNA expression of Smad7 was significantly decreased (*P* < 0.05; Fig. 5a) in miR-17-5p mimic group of cells compared to the scramble group of cells. Similarly, it was significantly increased (*P* < 0.01; Fig. 5a) in si-miR-17-5p group of cells compared to the siNC group of cells. Western blot analysis also revealed the same findings (Fig. 5b). Relative luciferase assay revealed that Smad7 promoter expression was significantly decreased (*P* < 0.05; Fig. 5c) in the miR-17-5p mimic group of cells.

### Suppression of miR-17-5p reduced cell injury by overexpression of Smad7

**Fig. 6:**
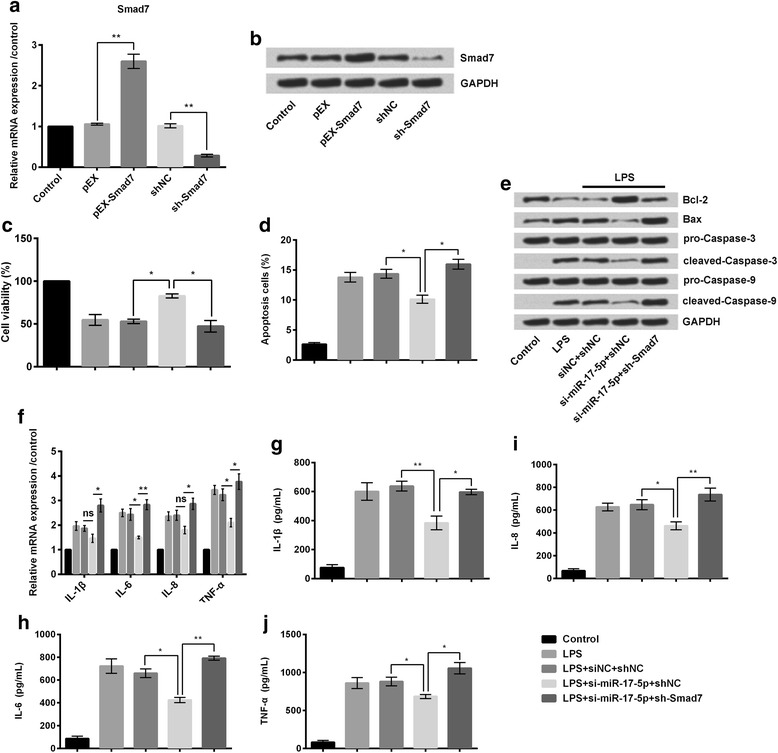
Following transfection of RPMI2650 cells with full length Smad7 sequence or short hairpin RNA directed against Smad7. **a** RT-PCR was done to estimate relative mRNA expression of Smad7 in different groups of cells; **b** Western blot analysis also supported the above findings; **c** Cell viability was significantly increased following suppression of miR-17-5p; however, it was significantly increased following suppression of both miR-17-5p and Smad 7 expressions despite treatment with LPS; **d** Apoptosis assay revealed that although suppression of miR-17-5p suppressed apoptosis, suppression of both miR-17-5p and Smad7 expressions led to promotion of apoptosis; **e** Western blot also supported the same. **f** Relative mRNA expressions of different inflammatory cytokines were decreased following suppression of miR-17-5p expression; however suppression of both miR-17-5p and Smad7, led to increased mRNA expression of different cytokines; **g-j** Elisa estimated exact amount of the said inflammatory markers. Smad7: Mothers against decapentaplegic homolog 7; LPS: Lipopolysaccharides. * *P* < 0.05; **P < 0.01

RT-PCR revealed that relative mRNA expression of Smad7 was significantly increased (*P* < 0.01; Fig. 6a) in the pEX-Smad7 group of cells (RPMI2650 cells transfected with full-length Smad7 sequences constructed in pEX-2 plasmid). Similarly, it was significantly decreased (*P* < 0.01; Fig. 6a) in the sh-Smad7 group of cells (RPMI2650 cells transfected with shRNA directed against Smad7). Western blot analysis also revealed the same findings (Fig. 6b). CCK-8 assay revealed that the percentage of viable cells was significantly increased (*P* < 0.05; Fig. 6c) following knockdown of miR-17-5p despite treatment with LPS in the LPS + si-miR-17-5p + shNC group of cells compared to the control group of cells treated with LPS (LPS + siNC+shNC). Again, suppression of both miR-17-5p and SMAD7 expressions led to significant increase (*P* < 0.05; Fig. 6c) in the percentage of viable cells despite treatment with LPS in the LPS + si-miR-17-5p + shSmad7 group of cells compared to the the LPS + si-miR-17-5p + shNC group of cells. Apoptosis assay revealed that the percentage of apoptotic cells was significantly decreased (*P* < 0.05; Fig. 6d) in the LPS + si-miR-17-5p + shNC group of cells compared to the control group of cells treated with LPS (LPS + siNC+shNC). Again, suppression of both miR-17-5p and SMAD7 expressions led to significant increase (*P* < 0.05; Fig. 6d) in apoptosis despite treatment with LPS in the LPS + si-miR-17-5p + shSmad7 group of cells compared to the the LPS + si-miR-17-5p + shNC group of cells Western blot analysis also revealed similar findings, as there were decrease in the amounts of pro-apoptotic factor Bax, cleaved-caspase-3, and cleaved-caspase-9, in LPS-treated si-miR-17-5p group of cells compared to other groups of cells, whereas suppression of expressions of both miR-17-5p and Smad7 revealed just the opposite results (Fig. 6e). Relative mRNA expressions of the different inflammatory cytokines namely, IL-1β, IL-6, IL-8, and TNF-α, were decreased following only suppression of miR-17-5p despite treatment with LPS (as in LPS + si-miR-17-5p + shNC group of cells) whereas suppression of both miR-17-5p and Smad7 expressions (as in LPS + si-miR-17-5p + shSmad7 group of cells) led to increased expressions of the said inflammatory cytokines (Fig. 6f). Elisa was done to estimate the amounts of the said inflammatory cytokines (Fig. 6g-j) released by the different groups of cells. Similar to Fig. 6e, it was found that the amounts of IL-1β (*P* < 0.01; Fig. 6g), IL-6 (*P* < 0.05; Fig. 6h), IL-8 (*P* < 0.05; Fig. 6i), and TNF-α (*P* < 0.05; Fig. 6j) released from the LPS-treated si-miR-17-5p group of cells were significantly lower compared to the LPS-treated control group of cells. Similarly, knockdown of both miR-17-5p and Smad7 as in the LPS + si-miR-17-5p + shSmad7 group of cells led to significant increase in the released amounts of IL-1β (*P* < 0.05; Fig. 6g), IL-6 (*P* < 0.01; Fig. 6h), IL-8 (*P* < 0.01; Fig. 6i), and TNF-α (*P* < 0.05; Fig. 6j).

Thereby, it was found that miR-17-5p aggravated LPS-induced injury of RPMI2650 cells by suppressing expression of Smad7.

### Overexpression of Smad7 alleviated LPS-induced cell injury

**Fig. 7:**
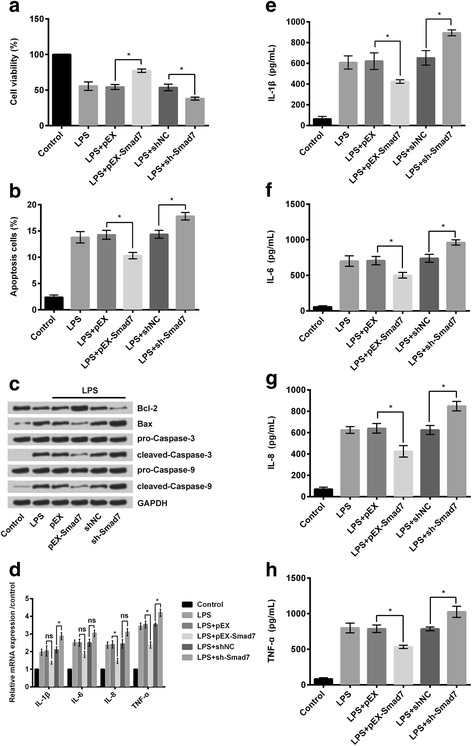
Overexpression of Smad7 protected RPMI2650 cells against LPS-induced injury. **a** Cell viability was significantly increased in cells overexpressing Smad7 and vice versa; **b** Apoptosis was also suppressed in cells overexpressing Smad7; **c** Western blot analysis of apoptosis related proteins (Bcl-2, Bax, cleaved-caspase-3, and cleaved-caspase-9) also showed similar findings; **d** RT-PCR revealed that relative mRNA expressions of the inflammatory cytokines were suppressed in cells overexpressing Smad7; **e-h** Elisa revealed that the amounts of the said inflammatory cytokines were decreased in cells overexpressing Smad7. LPS: lipopolysaccharide; Smad7: Mothers against decapentaplegic homolog 7; RT-PCR: quantitative real-time polymerase chain reaction. * *P* < 0.05

LPS-treated RPMI2650 cells overexpressing Smad7 as in (LPS + pEX-Smad7 group of cells) revealed significant increase (*P* < 0.05; Fig. 7a) in the percentage of viable cells and significant decrease in the percentage of apoptotic cells (*p* < 0.05; Fig. 7b) despite treatment with LPS. Similarly, suppression of Smad7 expression led to significant decrease (*P* < 0.05; Fig. 7a) in the percentage of viable cells and significant increase in the percentage of apoptotic cells (*p* < 0.05; Fig. 7b). Western blot also supported the above findings, as there were decrease in the amounts of pro-apoptotic factor Bax, cleaved-caspase-3, and cleaved-caspase-9, and increase in the amount of anti-apoptotic factor Bcl-2 in the cells overexpressing Smad7 despite treatment with LPS compared to other groups of cells, whereas suppression of expressions of Smad7 revealed just the opposite results (Fig. 7c). RT-PCR revealed that the Relative mRNA expressions of the different inflammatory cytokines namely, IL-1β, IL-6, IL-8, and TNF-α, were decreased following overexpression of Smad7 despite treatment with LPS (as in LPS + pEX-Smad7 group of cells) whereas suppression of Smad7 expressions (as in LPS+ shSmad7 group of cells) led to increased expressions of the said inflammatory cytokines (Fig. 7d). Similar to Fig. 7d, the amounts of IL-1β (*P* < 0.05; Fig. 7e), IL-6 (*P* < 0.05; Fig. 7f), IL-8 (*P* < 0.05; Fig. 7g), and TNF-α (*P* < 0.05; Fig. 7h) released from the cells overexpressing Smad7 (as in LPS + pEX-Smad7 group of cells) were significantly lower despite treatment with LPS compared to the LPS-treated control group of cells. Similarly, knockdown of Smad7 as in the LPS+ shSmad7 group of cells led to significant increase in the released amounts of IL-1β (*P* < 0.05; Fig. 7e), IL-6 (*P* < 0.05; Fig. 7f), IL-8 (*P* < 0.05; Fig. 7g), and TNF-α (*P* < 0.05; Fig. 7h).

Hence, it can be said that Smad7 protected RPMI2650 cells from LPS-induced injury.

### Smad7 overexpression and miR-17-5p suppression alleviated LPS-induced cell injury by inactivation of NF-κB and Wnt/β catenin pathways

**Fig. 8:**
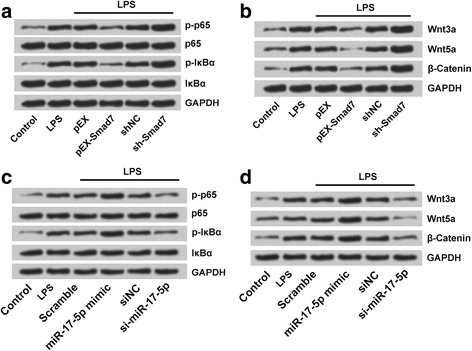
Smad7 overexpression and miR-17-5p suppression alleviated LPS-induced cell injury by inactivation of NF-κB and Wnt/β catenin pathways. Overexpression Smad7 led to inactivation of both **a** NF-κB, and **b** Wnt/β catenin pathways. Suppression miR-17-5p led to inactivation of both **c** NF-κB, and **d** Wnt/β catenin pathways

Western blot revealed that overexpression of Smad7 as in (LPS + pEX-Smad7 group of cells) led to inactivation of both NF-κB and Wnt/β catenin pathways (Fig. 8a-b). As there were decrease in the expression of NF-κB pathway associated proteins, namely phosphorylated p65 (p-p65) and phosphorylated INKα (p- INKα) in LPS + pEX-Smad7 group of cells (Fig. 8a) compared to the cells with suppressed Smad7 expression (LPS + sh-Smad7 group of cells). Similarly, Wnt/β catenin pathway associated proteins like Wnt3a, Wnt 5a, and β-Catenin were also decreased in cells overexpressing Smad7 as in pEX-Smad7 group of cells (Fig. 8b) compared to the cells with suppressed Smad7 expression (LPS + sh-Smad7 group of cells). Of contrast, miR-17-5p overexpression (LPS + miR-17-5p mimic) led to activation of NF-κB and Wnt/β catenin pathways, while miR-17-5p suppression (LPS + si-miR-17-5p group of cells) inactivated these two pathways (Fig. 8c-d).

## Discussion

Rhinitis, one of the most common chronic upper airway diseases, is quite prevalent across the world [1–3]. Accumulating evidences demonstrate that miRNAs are implicated in the pathogenesis and biological processes of many diseases [4]. miR-17-5p is one of the widely investigated miRNAs; however majority of the studies have explored its role in cancer [9, 10]. Again, several studies have discussed the role of miRNAs in the pathogenesis of rhinitis [6, 7]; however the role of miR-17-5p in the pathogenesis of rhinitis remains unclear. In this study we have explored the role of miR-17-5p in the pathogenesis of rhinitis and elucidated the underlying molecular mechanism using the RPMI2650 cell line. RPMI2650 is a human nasal epithelial cell line, with features resembling those of normal nasal epithelium cells [21].

LPS, cell wall component of Gram negative bacteria, has already been in used to establish in vitro rhinitis model [19]. Bae JS and colleagues have used LPS-induced rhinitis model for evaluation of role of IL-17 in the pathogenesis of rhinitis. Several studies have described that LPS treatment led to cell injury by suppression of cell proliferation, promotion of apoptosis and increased elicitation of inflammatory cytokines [19, 22]. Tang ZL, et al. in their study have described that LPS induced apoptosis in sheep pulmonary artery endothelial cells (SPAEC) and prior treatment of the SPAEC cells with NO-donor, S-nitroso-N-acetylpenicillamine (SNAP) protected them from LPS-induced apoptosis [23]. Qi J and colleagues have shown in their study that LPS treatment of murine macrophages led to increased released of inflammatory cytokines, namely IL-6, and TNF-α. They also demonstrated that overexpression of miR-210 led to suppression of LPS-induced release of the said inflammatory cytokines. Similar to these previous studies, our results demonstrated that LPS could lead to RPMI2650 cells injury, as inhibited cell viability, induced apoptosis and stimulated the secretion of IL-1β, IL-6, IL-8 and TNF-α. Besides, we found that LDH release was significantly increased in response to LPS stimulation. Since LDH release occurs when cells were damaged and injured, thus we inferred that cytokines might be released from necrotic cell. We cannot exclude the possibility that the release of cytokines also via exocytotic release. Further investigations are required. Previously studies have described that LPS-induced cell injury can be mediated by modulation of expression of miRNAs [24]. Fang et al. demonstrated that miR-1246 mediated LPS-induced lung injury, which was accompanied by reduction apoptosis and production of IL-1β and TNF-α [25]. Similarly, Wang W and colleagues described that miR-155 promoted LPS-induced acute lung injury in both mice and rat [26]. MiR-17-5p play an important role in a diverse range of cellular functions which has been reported in various diseases [12, 27]. Another study found that suppression of miR-17-5p could inhibit LPS-induced astroglial proliferation in vitro [28]. However, till date, no study is available exploring miR-17-5p expression in LPS-induced cell injury. In this study we found that the relative RNA expression of miR-17-5p was increased significantly in LPS-treated RPMI2650 cells, and overexpression of miR-17-5p significantly aggravated LPS-induced injury in RPMI2650 cell.

Smad7 is a protein encoded by the *SMAD7* gene [29]. Several studies have described the protective role of Smad7 in inflammatory diseases [29, 30]. Liu GX and his colleagues have described that Smad7 protected the kidneys from angiotensin II mediated inflammation in murine model [31]. Meanwhile, recent studies reported that Smad7 could enhance muscle differentiation and play an important role in prevent of cancer cell metastasis [32, 33]. However, whether Smad7 was involved in regulating LPS-induced cell injury in rhinitis remain unclear. In our study we found that suppression of Smad7 expression led to aggravation of LPS-induced cell injury, whereas overexpression of Smad7 alleviated LPS-induced injury of RPMI2650 cells.

NF-κB pathway is considered as the prototype pro-inflammatory pathway mainly because of its role on expression of cytokines, and chemokines [34]. Similar to our findings, Fei XJ and colleagues shown in their study that *Acanthopanax senticosus*, a common medicine in Oriental medicine protected murine lung cells from LPS-induced injury via inactivation of NF-κB pathway [35]. Furthermore, it was found that the protective action of Smad7 against LPS-induced cell damage is mediated by inactivation of NF-κB pathway as estimated by western blot. Similar to our findings, Wang J, et al. described that Smad7 inactivated NF-κB pathway and protected mice from hepatocarcinogenesis [36].

Wnt/βcatenin pathway is one of the evolutionarily conserved pathways. It plays important roles both in biological processes and in diseases [37]. LI B and colleagues demonstrated that mesenchymal stem cells protected alveolar macrophages from LPS-induced apoptosis by inhibiting Wnt/β catenin pathway [38]. Wu et al. found that Smad7 down-regulated Wnt4, Wnt5a, Wnt7a and Wnt10a expression in osteoarthritis [39]. Similar with these previous studies, our results demonstrated that Smad7 protected RPMI2650 cells from LPS-induced damage by inactivation of Wnt/β-catenin pathway. More interestingly, previous studies have proposed cross-regulation between the NF-κB and Wnt/β-catenin pathways [40, 41]. Cho et al., have indicated that diclofenac inhibited Wnt/β-catenin signaling in colon cancer cells through the activation of NF-κB [42]. However, is there exist correlation between Smad7 mediated Wnt/β-catenin and NF-κB signaling still need to be further revealed.

## Conclusions

Thus from our study it can be concluded that overexpression of miR-17-5p aggravated LPS-induced injury of RPMI2650 cells by negatively regulating the expression of Smad7, which protected the RPMI2650 cells via inactivation of NF-κB and Wnt/β-catenin pathway.

## Abbreviations

CCK-8: Cell Counting Kit-8
FBS: Fetal bovine serum
LPS: Lipopolysaccharide
miR-17-5p: microRNA-17-5p
Smad7: mothers against decapentaplegic homolog 7
TNF-α: Tumor necrosis factor α
